# Discovery of New 7-Propanamide Benzoxaborole as Potent Anti-SKOV3 Agent via 3D-QSAR Models

**DOI:** 10.3390/ijms27010472

**Published:** 2026-01-02

**Authors:** Liyang Ji, Jiong Zhang, Huchen Zhou, Yaxue Zhao

**Affiliations:** 1 School of Pharmaceutical Sciences, Shanghai Jiao Tong University, Shanghai 200240, China; lyji2020@sjtu.edu.cn; 2 Key Laboratory of Tropical Biological Resources of Ministry of Education and Hainan Engineering Research Center for Drug Screening and Evaluation, School of Pharmaceutical Sciences, Hainan University, Haikou 570228, China; 3 Inflammation and Immune Mediated Diseases Laboratory of Anhui Province, School of Pharmacy, Anhui Medical University, Hefei 230032, China; zhangjiong@ahmu.edu.cn

**Keywords:** benzoxaboroles, SKOV3, 3D-QSAR, anti-cancer

## Abstract

Benzoxaboroles have garnered significant interest for their therapeutic potential in various diseases. Among them, 7-propanamide benzoxaborole has served as a new and valuable chemotype for anti-cancer agents, although their definitive intracellular target(s) remains elusive. Herein, three-dimensional quantitative structure–activity relationship (3D-QSAR) was used to systematically investigate the structure–activity relationships (SAR) of a series of 7-propanamide benzoxaboroles. Comparative molecular field analysis (CoMFA, r^2^ = 0.991, q^2^ = 0.626) and comparative molecular similarity indices analysis (CoMSIA, r^2^ = 0.964, q^2^ = 0.605) revealed critical structural determinants of 7-propanamide benzoxaboroles for inhibition of the ovarian cancer cell (SKOV3) proliferation. Based on the guidance of the critical structural determinants, we designed a new benzoxaborole compound **42** with high predicted inhibition activity values. In vitro proliferation assessment showed that compound **42** exhibited superior inhibitory potency to lead compound **1** and comparable activity to compound **41**. These findings indicated that the SAR of benzoxaborole compounds through 3D-QSAR can offer valuable theoretical insights for the structural optimization of new benzoxaboroles as anti-SKOV3 agents.

## 1. Introduction

**Figure 1:**
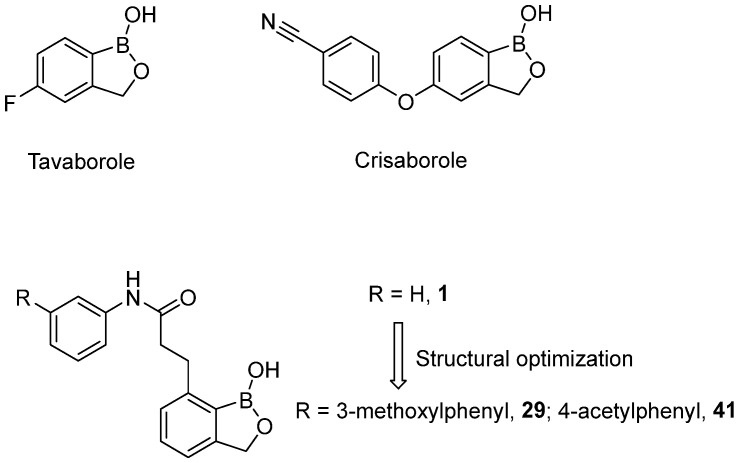
Structures of tavaborole (Kerydin), crisaborole (Eucrisa), compounds **1**, **29** and **41**.

Benzoxaboroles are a class of boron-containing heterocyclic compounds with unique chemical structures and biological activity. They have received extensive attentions for their broad applications in drug discovery, particularly in the fields of anti-cancer, anti-inflammatory, and anti-infective therapies [1,2]. To date, there are two approved benzoxaborole medications. In 2014, tavaborole (Kerydin, Figure 1) was authorized for the treatment of onychomycosis. Meanwhile, in 2016, crisaborole (Eucrisa, Figure 1) obtained approval for addressing mild to moderate atopic dermatitis.

Our research has focused on the anti-cancer and anti-infective applications of benzoxaborole compounds [3,4,5,6,7]. In 2019, we reported the discovery of 7-propanamide benzoxaboroles as potent anti-cancer agents [7]. These compounds showed inhibitory potency against multiple cancer cell lines, with the highest potency observed for the SKOV3 cell line. The influence of different substitutions on the phenyl ring of the lead compound **1** regarding their anti-SKOV3 activity was investigated by us, among which the most potent analog **41** (Figure 1) showed significant inhibitory activity in both in vitro cell proliferation tests and in vivo tumor xenograft growth experiments. We investigated their potential apoptosis mechanisms and found that compound **29** (Figure 1) can induce apoptosis by triggering the cleavage of poly(ADP-ribose) polymerase (PARP) and caspase-3/9. However, although there are several studies on the cellular targets of benzoxaborole, such as phosphodiesterase 4 (PDE4) [8], leucyl-tRNA synthetases (LeuRS) [9] and cleavage and polyadenylation specificity factor 3 (CPSF3) [10], the definitive target(s) of our anti-cancer compounds remains elusive and calls for more in-depth investigation.

The three-dimensional quantitative structure–activity relationship (3D-QSAR) is an effective computational approach in computer-aided drug design (CADD), establishing mathematical models between molecular descriptors and biological activity to guide rational drug optimization [11,12,13]. The most frequently utilized techniques for developing 3D-QSAR are comparative molecular field analysis (CoMFA) and comparative molecular similarity indices analysis (CoMSIA). Here, to elucidate structure–activity relationships (SAR) of our reported benzoxaborole derivatives, we established 3D-QSAR models, specifically CoMFA and CoMSIA, to guide molecular optimization. Subsequently, we designed and synthesized a new compound **42** with high predicted activity value based on the obtained contour maps derived from the 3D-QSAR model, which has been validated in subsequent experimental assay. This study provides a valuable theoretical foundation for predicting activity and guiding structural optimization of benzoxaborole as anti-SKOV3 agents.

## 2. Results and Discussion

### 2.1. 3D-QSAR Study

#### 2.1.1. Acquisition of Conformations

The conformation generation was performed for all the 41 compounds to enable subsequent 3D-QSAR modeling. Based on the known bioactive conformation of benzoxaboroles, the B atom adopts a tetrahedral anionic form [8,9,10]. Therefore, we first conducted structural refinement of all small molecules under physiological conditions to more accurately simulate their binding behavior with the target site. Then all compounds underwent conformational sampling to generate diverse, low-energy 3D structures. For each compound, 50 conformers were generated and subsequently clustered into three groups based on structural similarity. The conformer closest to the known bioactive conformation was selected as the representative conformer for subsequent 3D-QSAR model construction (alternate conformers were also included in 3D-QSAR modeling, with statistical parameters detailed in Tables S1 and S2).

#### 2.1.2. Molecular Alignment

**Figure 2:**
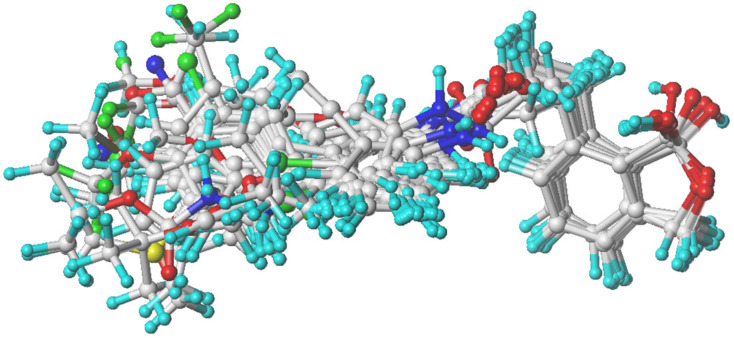
Molecular alignment based on their representative conformations in SYBYL-X (all 41 compounds).

The effectiveness of 3D-QSAR models is reliant on the alignment of molecules, as the data on biological activity are closely associated with various substitutions at a particular position within the same series of compounds. Based on their representative conformations, the molecular alignment of all compounds was shown in Figure 2.

#### 2.1.3. 3D-QSAR Statistics

The models of 7-propanamide benzoxaboroles based on 3D-QSAR, specifically CoMFA and CoMSIA, were constructed by SYBYL-X, as shown in Table 1. Table 2 provided a summary of all the statistical parameters of the 3D-QSAR models.

The CoMFA model was developed based on two fields: steric (S) and electrostatic (E). This model produced a cross-validated coefficient q^2^ of 0.626 with an optimal component count of 8, standard error of estimate (SEE) of 0.090, coefficient of determination (r^2^) value of 0.991 and F-test value (F) of 327.630. The contributions of the parameters in the corresponding fields were 72.9% from the field S and 27.1% from the field E descriptor, suggesting that the field S had a greater influence.

The CoMSIA model incorporated five distinct molecular interaction fields: S, E, hydrophobic (H), hydrogen bond donor (D) and acceptor (A). CoMSIA-SEDA was proven to be the best model, which was employed for detailed analysis, giving a cross-validated coefficient q^2^ of 0.605 with an optimal component of 6, SEE value of 0.173, r^2^ of 0.964 and F value of 116.389. The contributions of the parameters in the corresponding fields were 16.3%, 22.8%, 22.2%, and 38.7% for S, E, D, and A fields, respectively. It could be found that the field A made great contributions for the SKOV3 inhibitory activity especially.

#### 2.1.4. Validation of the 3D-QSAR Models

In addition, the external predictability was further validated using a test set of 8 compounds and their activity values were predicted by the 3D-QSAR models. As shown in Table 1, it could be found that the predicted pIC_50_ values agreed well with the experimental values in a tolerable error range. As shown in Table 2, the CoMFA model yielded a predictive correlation coefficient (r^2^_pred_) of 0.941, accompanied by a modified term (r^2^_m_) of 0.796 and an SDEP_ext_ value of 0.260. In contrast, the CoMSIA model achieved an r^2^_pred_ value of 0.961, accompanied by r^2^_m_ value of 0.919 and SDEP_ext_ value of 0.308. For external compounds, the CoMSIA model showed a superior ability to predict the activity of external compounds compared to CoMFA.

**Figure 3:**
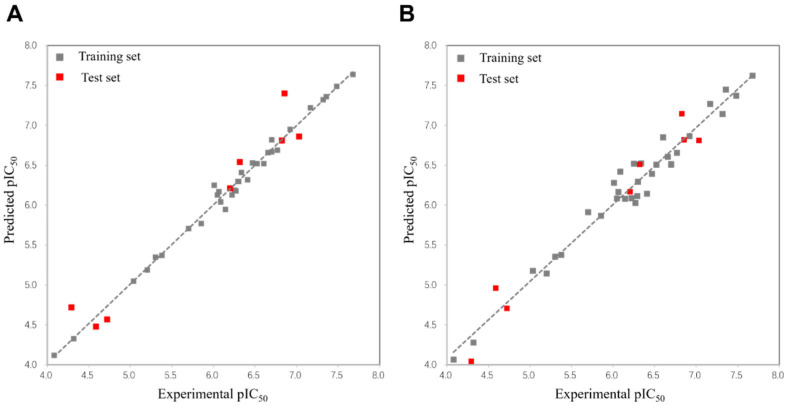
The scatter plots illustrating the exp. versus pred. bioactivity values (pIC_50_) for the CoMFA model (**A**) and the CoMSIA model (**B**), respectively.

For the studied benzoxaborole derivatives, the scatter plots of the experimental versus predicted biological activity values were shown in Figure 3. It was evident that both sets of values for nearly all compounds clustered around the Y = X line, indicating that the predicted results closely aligned with the experimental ones and demonstrating the model’s strong predictive capability.

#### 2.1.5. Analysis of Contour Maps

**Figure 4:**
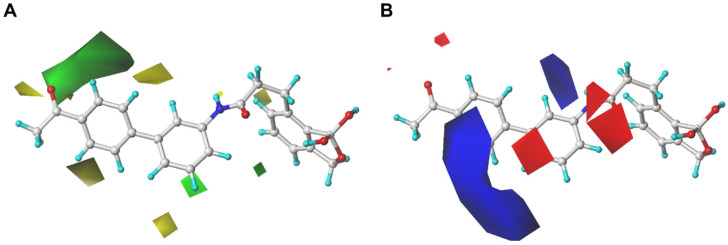
Contour maps of fields S (**A**) and E (**B**) of the CoMFA model based on compound **41**. Sterically favored areas for enhancing activity of anti-SKOV3 were indicated by green areas, while sterically unfavored areas were indicated by yellow areas. Electron-donating groups favored areas which might be beneficial for enhancing activity of anti-SKOV3 were indicated by blue areas, while electron-withdrawing groups favored areas were indicated by red regions.

**Figure 5:**
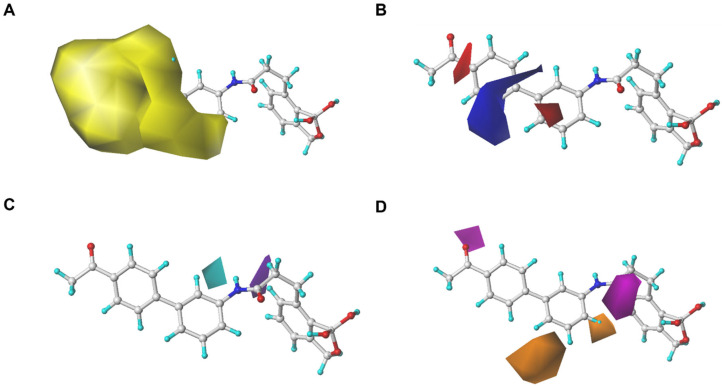
Contour maps of fields S (**A**), E (**B**), D (**C**) and A (**D**) of the CoMSIA model based on compound **41**. Sterically unfavored areas were indicated by yellow areas. Electron-donating groups favored region were indicated by blue areas, while electron-withdrawing groups favored areas were indicated by red areas. Hydrogen bond donor groups favored region were indicated by cyan area, while unfavored area was indicated by the purple area. Hydrogen bond acceptor groups were indicated by magenta areas, while hydrogen bond acceptor groups unfavored areas were indicated by orange areas.

The SAR between benzoxaborole compounds and their activity can be effectively shown by displaying the QSAR equation using three-dimensional contour maps. As shown in Figure 4 and Figure 5, compound **41**, which demonstrated the strongest anti-SKOV3 activity, was chosen as a template to profile the results of the CoMFA and CoMSIA models.

Figure 4A illustrated the contour map of the steric field from CoMFA. In this representation, green and yellow areas signified the impact of the steric field on biological activity. Specifically, the green areas surrounding the compound suggested that groups with greater spatial connectivity might improve the compound’s activity, whereas the yellow areas implied that these groups might reduce activity. From Figure 4A, we can see that the green region covers the meta- and para-positions on the terminal benzene ring of the compound, indicating that incorporation of a slightly bigger group in both positions would be beneficial for enhancing the compound’s activity. For example, the biological activity of compound **30** (pIC_50_ = 7.32), which has the R_7_ substituent of a 4-methoxy group, was significantly higher than compound **23** (pIC_50_ = 6.60). Also, it is worth noting that multiple substituent effects may be contributing simultaneously, not solely steric factors. The electrostatic field contour map for CoMFA was shown in Figure 4B, demonstrating how the electrostatic field influenced activity, as represented by blue and red areas. The blue regions surrounding the compound suggested that the incorporation of an electron-donating group would potentially improve the compound’s activity. From Figure 4B, we can see that the blue region covered the meta-position on the terminal benzene ring of the compound, indicating that introducing an electron-donating group in this position might enhance the compound’s activity. For example, compound **29** (pIC_50_ = 7.48), which has the R_8_ substituent of 3-methoxyphenyl, showed significantly higher biological activity than that of compound **23** (pIC_50_ = 6.60).

Figure 5A illustrated the contour map of the steric field from CoMSIA. Unlike the CoMFA model predictions, introducing a bulky group at the central benzene ring of 7-propanamide benzoxaboroles might lead to reduced activity. Considering the fundamental differences in the modeling principles between CoMFA and CoMSIA, this complementary behavior reflects the inherent strengths of each method in addressing different aspects of SARs. The CoMFA method is well-suited for guiding precise local modifications, whereas CoMSIA can capture broader property distributions, making it more appropriate for analyzing large-scale molecular trends. For example, compound **21** (pIC_50_ = 5.85) with a naphthalene substituent showed significantly lower biological activity compared to compound **1** (pIC_50_ = 6.70). The contour map of the electrostatic field of CoMSIA was shown in Figure 5B. The red regions around the para-position of both the central and terminal benzene rings suggested that introducing an electron-withdrawing group at these positions might enhance the compound’s activity. For example, compound **26** (pIC_50_ = 7.03) with an R_7_ fluorine substituent showed better biological activity than compound **23** (pIC_50_ = 6.60). Additionally, the blue region near the ortho- and meta-positions on the terminal benzene ring suggested that introducing an electron-donating group at both positions might enhance activity, similar to the pattern seen in the CoMFA model. The hydrogen bond donor field’s contour map for CoMSIA was shown in Figure 5C. The cyan area signified locations where the addition of a hydrogen bond donor group might potentially improve the compound’s activity, whereas the purple area highlighted region where such an addition might reduce the compound’s activity. The findings suggested that the hydrogen atom attached to the nitrogen within the amide bond had the potential to act as a hydrogen bond donor, thus strengthening the interaction between these inhibitors and their target. The contour map illustrating the hydrogen bond acceptor field of CoMSIA was shown in Figure 5D. Magenta areas highlighted the locations where the incorporation of a hydrogen bond acceptor group might potentially improve the activity, whereas orange areas signified regions where such groups were less favorable. As shown in Figure 5D, a magenta area was located close to the R_7_ position on the terminal benzene ring of the compound, suggesting that the compound’s activity might be enhanced by adding a hydrogen bond acceptor in this area. For example, compound **41** (pIC_50_ = 7.68), with an acetyl group as the R_7_ substituent, showed significantly higher biological activity than compound **23** (pIC_50_ = 6.60).

#### 2.1.6. SAR Summary

**Figure 6:**
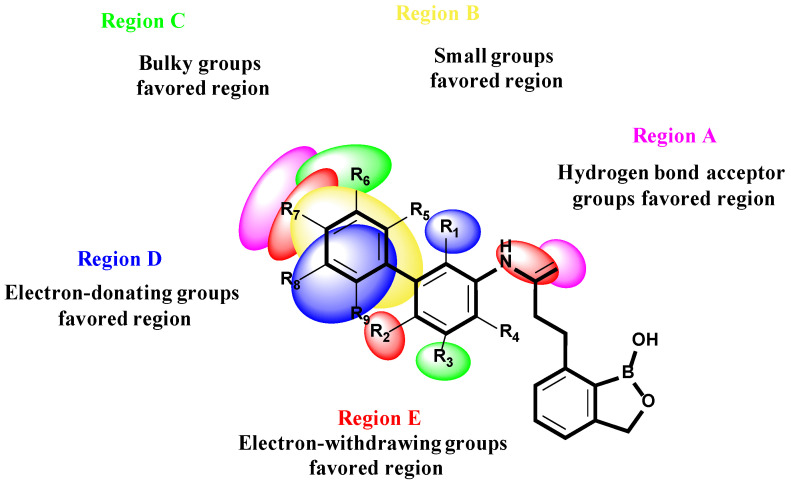
SAR diagram of 7-propanamide benzoxaboroles as potent anti-SKOV3 agents.

We obtained the SAR diagram of 7-propanamide benzoxaboroles as SKOV3 inhibitors based on the outcome of CoMFA and CoMSIA analysis. As shown in Figure 6, in region A, the introduction of a hydrogen bond acceptor group might be beneficial for enhancing the activity, such as the carbonyl group [14]. In region B, the introduction of a small group might be beneficial for enhancing the activity, such as the methyl group [15]. In region C, the introduction of a slightly sterically group might be beneficial for enhancing the activity, such as the methoxy group [16]. In Region D, the introduction of an electron-donating group might be beneficial for enhancing the activity, such as the methoxy group [17]. In region E, the introduction of an electron-withdrawing group might be beneficial for enhancing the activity, such as trifluoromethyl [18]. It is worth noting that, the identified SAR trends offer valuable guidance for the current dataset, and their applicability to other chemical series might be limited.

### 2.2. Design of New Anti-SKOV3 Agent

According to the SAR obtained from 3D-QSAR model, one unknown SKOV3 inhibitor featuring the 7-propanamide benzoxaborole scaffold was designed by incorporating different substituents on the central phenyl ring of compound **41**, as shown in Table 3. Compound **42** was designed by introducing an electron-donating carbamoyl group at the meta-position of the central benzene ring to replace the biphenyl linkage, with a small-sized propenyl group serving as a small substituent. Using the built CoMFA and CoMSIA models, the pIC_50_ value of the designed compound was predicted. As shown in Table 3, compound **42** showed satisfactory predictive inhibitory activity against SKOV3 cells compared to compound **41**, and the predictive values aligned with the summarized SAR.

**Scheme 1:**
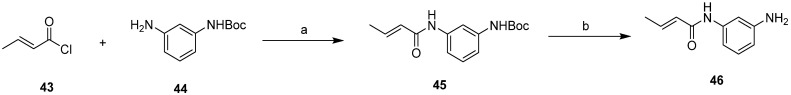
Synthesis of amine intermediates. Reagents and conditions: (a) TEA, DCM; (b) CF_3_COOH, DCM.

**Scheme 2:**
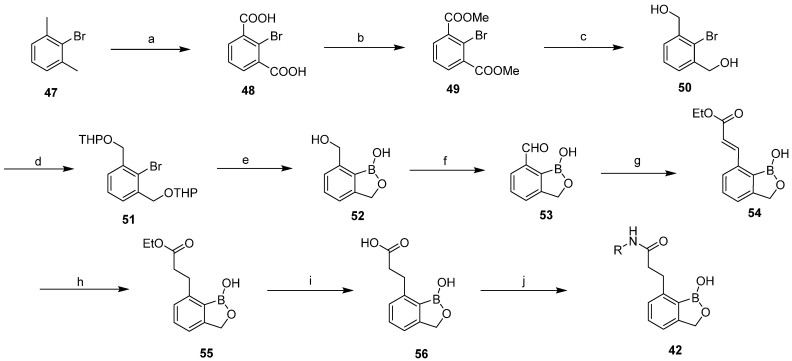
Synthesis of 7-propanamide benzoxaborole **42**. Reagents and conditions: (a) KMnO_4_, t-BuOH, H_2_O, 70 °C; (b) SOCl_2_, reflux, TEA, MeOH; (c) LiBH_4_, MeOH, THF; (d) DHP, p-TsOH, DMF; (e) n-BuLi, B(i-PrO)_3_, THF, −78 °C to rt, 6 M HCl, THF, MeOH; (f) PCC, DCM; (g) Ph_3_P=CHCOOCH_2_CH_3_, toluene; (h) Pd/C, H_2_, EtOH; (i) NaOH, MeOH, H_2_O; (j) RNH_2_, HATU, TEA, DCM.

To confirm the predicted inhibitory activity of compound **42**, it was synthesized and tested in vitro. The corresponding amines were synthesized according to the method outlined in Scheme 1. Aniline **46** was obtained by acid-catalyzed deprotection of the Boc-protected aniline intermediate **45**. Compound **45** was prepared by condensation of acyl chloride **43** with meta-phenylenediamine **44**. The 7-propanamide benzoxaborole derivative was prepared according to the method outlined in Scheme 2. According to the method we previously reported, the synthesis of the co-intermediate **56** was prepared [19]. Subsequent condensation with corresponding aniline **46** afforded the 7-propanamide benzoxaborole **42**. As shown in Table 3, in vitro proliferation assessment demonstrated that the experimental inhibitory activity data of compound **42** aligned well with its predicted value, supporting the 3D-QSAR model’s reliability for guiding structural optimization. However, while the results are encouraging, the conclusions regarding the predictive power of the model will be stronger if more than one designed compound were synthesized and tested. And this is a direction of our future work.

## 3. Materials and Methods

### 3.1. 3D-QSAR Construction

#### 3.1.1. 3D-QSAR Modeling Dataset

A set of 41 7-propanamide benzoxaboroles which were previously reported by our group [7] was used here. Their biological activity data against SKOV3 cells were measured under identical experimental conditions to ensure data comparability. In order to harmonize the activity scales, enhance the data linearity and improve the predictive capability of 3D-QSAR models, the IC_50_ values were transformed into pIC_50_ values using the formula pIC_50_ = −log_10_(IC_50_), which was utilized as the dependent variable for the SAR analysis. The range of pIC_50_ values for the identified compounds extends from 4.08 to 7.68, covering a broad and evenly distributed set of activity data, which provides a favorable dataset for the 3D-QSAR study.

#### 3.1.2. Generation of Conformational Ensembles

The LigPrep module [20] from Schrödinger (Schrödinger, LLC, New York, NY, USA) was utilized to generate 3D structures for all compounds, and the ionization states were assessed using Epik [21] at a pH of 7.4 ± 2.0. Then, conformational sampling was performed using the ConfGen module [22], generating 50 conformers per compound. Conformational clustering was performed using the Conformer Cluster module [23]. Clustering based on atomic root-mean-square deviation (RMSD) employed heavy atoms plus hydroxyl/sulfhydryl hydrogens (OH/SH) as the comparison regions, with the average linkage method applied to partition the 50 conformers of each compound into three distinct clusters. A representative conformer was selected from each cluster and saved for subsequent 3D-QSAR model construction.

#### 3.1.3. Molecular Alignment

In this study, conformational sampling and clustering were used to simulate the conformations of the compounds and obtain their alignment. As a result, all compounds were properly aligned for developing 3D-QSAR models. Then their 3D conformations were saved, imported into SYBYL-X (Tripos Inc., St. Louis, MO, USA) and re-aligned to ensure that their relative coordinate positions remained unchanged.

#### 3.1.4. CoMFA and CoMSIA Model Building

To generate the 3D-QSAR model, a random approximately 4:1 division of the 41 compounds into training and test sets was performed. The selection considered the uniform distribution of activity data and the structural diversity of the compounds to ensure the model’s general applicability. Finally, the training set comprised 33 compounds, with the remaining 8 serving as the test set.

The descriptors for CoMFA and CoMSIA were created by positioning the stacked compounds in a three-dimensional cubic lattice, utilizing a grid spacing of 2 Å. Within the CoMFA model, the steric and electrostatic field energies at every lattice point were determined through the application of Lennard-Jones and Coulomb potentials, employing sp^3^ hybridized carbon as the probe atom. This approach directly computes physical interaction energies but is sensitive to molecular alignment due to the singularities in these potential functions at short distances, thus providing superior spatial resolution and fragmented contours. Conversely, the molecular field energy function in the CoMSIA model took the form of a smoothed distance-dependent Gaussian function. This fundamentally different formulation avoids the mathematical singularities of CoMFA while maintaining sensitivity to molecular features, making it more suitable for large-scale similarity analysis. The contributions of fields (H, D, A) were derived from the probe atoms but also evaluated through Gaussian-type similarity indices rather than physical potentials, making the contributions less dependent on precise molecular alignment.

The relationship between the CoMFA and CoMSIA domains and their biological activity was examined through the partial least squares technique [24]. The leave-one-out (LOO) method [25] was utilized for cross-validation, yielding the cross-validation correlation coefficient (q^2^) and determining the optimal number of components (N). Furthermore, the statistical validity of the models was evaluated by employing the coefficient of determination (r^2^), the standard error of estimate (SEE), and the probability value (F).

#### 3.1.5. 3D-QSAR Model Validation

The ability of the 3D-QSAR models to predict was evaluated using an external test set consisting of 8 compounds. These compounds in the test set were also optimized and aligned as described earlier, and their activity was predicted using the models that were developed. To assess the model performance, the modified r^2^ term (r^2^_m_), the coefficient of determination for external validation (r^2^_pred_), the external standard deviation of prediction error (SDEP_ext_), and the domain of applicability were calculated.

### 3.2. Experimental Validation

#### 3.2.1. Chemistry

All solvents and reagents were obtained from Merck (Darmstadt, Germany) and Titan Ltd. (Shanghai, China) and utilized without additional purification unless specified otherwise. Column chromatography was conducted using Greagent silica gel with a mesh size of 200–300 (Titan Ltd., Shanghai, China). NMR spectra were recorded using Bruker Advance III spectrometers operating at 400 MHz (Bruker, Billerica, MA, USA). Chemical shifts (*δ*) are reported in parts per million (ppm) relative to the residual solvent, which served as an internal standard. High-resolution mass spectrometry (HRMS) data were acquired with an Agilent 6530 accurate-mass quadrupole time-of-flight LC/MS system (Agilent, Santa Clara, CA, USA). High-performance liquid chromatography (HPLC) was carried out on an Agilent 1200 system, utilizing a flow rate of 1 mL/min and a gradient of 10% MeOH/90% H_2_O to 100% MeOH over a 20 min period, employing a diode array detector. An Agilent Eclipse XDB-C18 column (4.6 mm × 150 mm, 3.5 μm) was utilized for the analysis. The purity of compounds evaluated for biological testing was determined based on the integrated UV chromatogram at 254 nm, with all compounds exhibiting a purity of ≥95%.

Tert-butyl(E)-(3-(but-2-enamido)phenyl)carbamate (**45**). A solution containing (E)-but-2-enoyl chloride (0.75 g, 7.17 mmol) and tert-butyl (3-aminophenyl)carbamate (1.24 g, 5.95 mmol) in dry DCM (20 mL) had TEA (0.85 g, 8.40 mmol) added rapidly while maintained in an ice bath. Following overnight stirring at room temperature, the resulting mixture was concentrated under vacuum. The residue was subsequently diluted with 30 mL of water and underwent extraction with DCM (3 times × 20 mL). The combined organic extracts were then dried using anhydrous Na_2_SO_4_, followed by filtration and concentration under vacuum. The crude residue underwent purification through silica gel chromatography, utilizing a petroleum ether/EtOAc gradient of 10:1 to 2:1 *v*/*v*, yielding compound **45** as a white solid (0.87 g, 52.3%). ^1^H NMR (400 MHz, CDCl_3_): δ 7.69 (s, 1H), 7.30 (d, J = 7.9 Hz, 1H), 7.22 (t, J = 8.0 Hz, 1H), 7.20–7.10 (m, 1H), 7.06 (d, J = 8.5 Hz, 1H), 6.97 (dq, J = 14.0, 6.9 Hz, 1H), 6.52 (s, 1H), 5.90 (dd, J = 15.1, 1.8 Hz, 1H), 1.91 (dd, J = 6.9, 1.6 Hz, 3H), 1.51 (s, 9H) ppm.

(E)-N-(3-aminophenyl)but-2-enamide (**46**). A solution of compound 45 (0.75 g, 2.71 mmol) in dry DCM (10 mL) had tri-fluoroacetic acid (3 mL) added dropwise while maintained in an ice bath. The mixture was stirred overnight at room temperature and then concentrated under vacuum. The resulting residue was gently quenched through the dropwise addition of a saturated aqueous NaHCO_3_ solution until a pH of 7.0 was achieved. The aqueous phase was then extracted with EtOAc (3 × 25 mL). The combined organic extracts were dried using anhydrous Na_2_SO_4_, filtered, and subsequently concentrated under vacuum. The crude residue underwent purification via silica gel chromatography (petroleum ether/EtOAc = 5:1 to 1:1 *v*/*v*), yielding compound **46** as a white solid (0.42 g, 87.0%). ^1^H NMR (400 MHz, CDCl_3_): δ 7.23 (s, 1H), 7.06 (t, J = 8.0 Hz, 1H), 6.96 (dq, J = 14.0, 6.8 Hz, 1H), 6.71 (d, J = 8.0 Hz, 1H), 6.42 (dd, J = 8.0, 2.2 Hz, 1H), 5.93 (dd, J = 15.2, 2.2 Hz, 1H), 3.48 (s, 2H), 1.89 (dd, J = 6.7, 1.8 Hz, 3H) ppm.

(E)-N-(3-(3-(1-hydroxy-1,3-dihydrobenzo[c][1,2] oxaborol -7-yl) propanamido)phenyl)but-2-enamide (**42**). A solution containing compound **56** (41 mg, 0.20 mmol) in anhydrous DCM (2 mL) was treated with compound **46** (68 mg, 0.38 mmol), HATU (182 mg, 0.48 mmol), and TEA (81 mg, 0.80 mmol). The mixture was stirred at room temperature overnight, after which it was subjected to vacuum evaporation. The resulting residue underwent purification through silica gel chromatography using a solvent system of DCM/MeOH in a gradient from 150:1 to 50:1 *v*/*v*, yielding compound **42** as a white solid (63 mg, 86.3%). ^1^H NMR (400 MHz, MeOD): δ 7.83 (d, J = 2.2 Hz, 1H), 7.35 (t, J = 7.6 Hz, 1H), 7.33–7.28 (m, 1H), 7.28–7.24 (m, 1H), 7.24–7.20 (m, 1H), 7.18 (t, J = 6.9 Hz, 2H), 6.94 (dd, J = 4.0, 1.7 Hz, 1H), 6.87 (t, J = 2.1 Hz, 1H), 6.10 (dd, J = 4.0, 2.6 Hz, 1H), 5.04 (s, 2H), 3.91 (s, 3H), 3.17 (t, J = 7.7 Hz, 2H), 2.69 (t, J = 7.7 Hz, 2H) ppm; ^13^C NMR (101 MHz, MeOD): δ 172.48, 161.26, 154.28, 145.07, 138.99, 138.77, 130.85, 128.75, 128.47, 126.80, 125.45, 118.68, 116.50, 115.62, 113.62, 112.82, 106.99, 70.77, 38.62, 35.53, 30.12 ppm; HRMS (ESI): [M + Na]^+^ C_21_H_20_BN_2_NaO_4_ calcd 387.1487 found 387.1489; HPLC: purity 98.1%, retention time 15.5 min.

#### 3.2.2. Cell Culture

The human ovarian cancer cell line SKOV3 was sourced from the American Type Culture Collection (ATCC, Manassas, VA, USA). These cancer cells were propagated in McCoy’s 5A complete medium (Gibco, Thermo Fisher Scientific, Waltham, MA, USA), which includes 10% fetal bovine serum (VivaCell, VivaCell Biosciences, Shanghai, China), 100 units/mL of penicillin, and 100 mg/mL of streptomycin (Gibco). Cultivation took place in an incubator maintaining a humidified environment with 5% CO_2_ at 37 °C.

#### 3.2.3. In Vitro Proliferation Assessment

The biological activity of compound **42** against SKOV3 was assessed using the CellTiter-Glo^®^ (Promega Corporation, Madison, WI, USA) assay. Following a 24 h incubation period, the cells were seeded in 96-well plates and treated with varying concentrations of the compounds for 72 h. After treatment, 50 μL of CellTiter-Glo^®^ Reagent solution was introduced to each well. The plates were left to incubate at room temperature for 10 min. The optical density was measured at 490 nm using a SpectraMax Paradigm enzyme marker (Molecular Devices, San Jose, CA, USA). Each compound was evaluated in triplicate wells for all concentrations. Data analysis was conducted using GraphPad Prism version 8.0 (GraphPad Software, LLC, San Diego, CA, USA).

## 4. Conclusions

In this study, 3D-QSAR techniques were utilized to investigate the SAR of benzoxaborole analogs. The models generated from CoMFA (q^2^ = 0.626, r^2^ = 0.991, r^2^_m_ = 0.796, r^2^_pred_ = 0.941) and CoMSIA (q^2^ = 0.605, r^2^ = 0.964, r^2^_m_ = 0.919, r^2^_pred_ = 0.961) demonstrated satisfactory statistical outcomes. Results showed that the constructed models exhibited robust internal and external predictive abilities. The generated contour maps elucidated the SAR of 7-propanamide benzoxaboroles and provided a clear explanation for the activity of the selected compounds. Based on the contour map results, introducing hydrogen bond acceptor groups in Region A, small groups in Region B, sterically groups in Region C, electron-donating groups in Region D, and electron-withdrawing groups in Region E could enhance the activity of the compounds. Guided by these structural insights for SKOV3 inhibition, we designed and synthesized a new compound **42** with high predicted inhibitory activity. In vitro proliferation assessment showed that compound **42** exhibited superior inhibitory potency than lead compound **1** and comparable activity to lead compound **41**. Experimental validation confirmed the computational predictions, demonstrating excellent agreement between theoretical and experimental values. In conclusion, the developed 3D-QSAR model offers theoretical direction for the structural enhancement of benzoxaborole derivatives in their role as effective anti-SKOV3 agents.

## Figures and Tables

**Table 1:** Experimental (Exp.), predicted (Pred.) pIC_50_ values and corresponding residuals (Res.) of 7-propanamide benzoxaboroles as determined by the 3D-QSAR (CoMFA and CoMSIA) model.

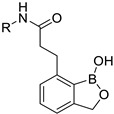						
Comp.	R	pIC_50_	CoMFA		CoMSIA	
		Exp.	Pred.	Res.	Pred.	Res.
Training Set						
**1**	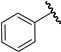	6.70	6.67	−0.03	6.52	−0.18
**2**	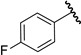	6.14	5.95	−0.19	6.08	−0.06
**3**	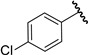	5.30	5.35	0.05	5.35	0.05
**4**	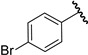	5.04	5.05	0.01	5.18	0.14
**6**	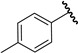	6.07	6.17	0.10	6.17	0.10
**7**	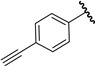	5.20	5.19	−0.01	5.14	−0.06
**8**	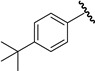	5.38	5.37	−0.01	5.38	0.00
**9**	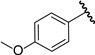	6.05	6.13	0.08	6.08	0.03
**10**	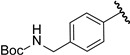	5.70	5.71	0.01	5.91	0.21
**11**	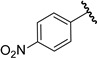	4.08	4.12	0.04	4.06	−0.02
**12**	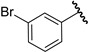	6.27	6.18	−0.09	6.03	−0.24
**14**	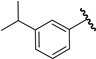	6.29	6.30	0.01	6.11	−0.18
**15**	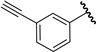	6.01	6.25	0.24	6.28	0.27
**16**	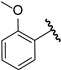	6.70	6.82	0.12	6.51	−0.19
**17**	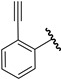	6.09	6.04	−0.05	6.42	0.33
**18**	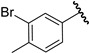	6.22	6.13	−0.09	6.09	−0.13
**20**	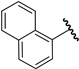	6.77	6.69	−0.08	6.66	−0.11
**21**	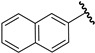	5.85	5.77	−0.08	5.86	0.01
**23**	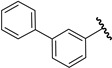	6.60	6.52	−0.08	6.85	0.25
**24**	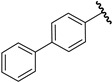	4.32	4.33	0.01	4.28	−0.04
**27**	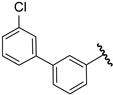	6.52	6.52	0.00	6.51	−0.01
**28**	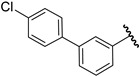	6.66	6.66	0.00	6.61	−0.05
**29**	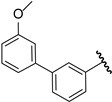	7.48	7.49	0.01	7.37	−0.11
**30**	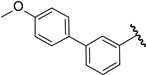	7.32	7.32	0.00	7.14	−0.18
**31**	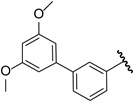	7.36	7.36	0.00	7.45	0.09
**32**	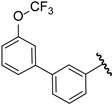	6.34	6.41	0.07	6.52	0.18
**33**	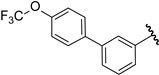	6.25	6.19	−0.06	6.52	0.27
**36**	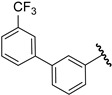	6.41	6.32	−0.09	6.14	−0.27
**37**	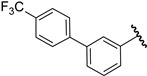	6.30	6.30	0.00	6.29	−0.01
**38**	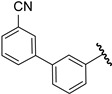	6.92	6.95	0.03	6.86	−0.06
**39**	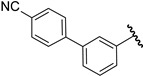	6.47	6.53	0.06	6.39	−0.08
**40**	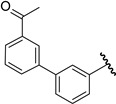	7.17	7.22	0.05	7.27	0.10
**41**	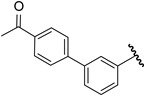	7.68	7.64	−0.04	7.62	−0.06
Test Set						
**5**	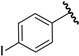	4.29	4.72	0.43	4.04	−0.25
**13**	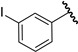	6.20	6.21	0.01	6.17	−0.03
**19**	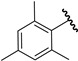	4.72	4.57	−0.15	4.71	−0.01
**22**	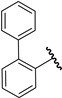	4.58	4.48	−0.10	4.96	0.38
**25**	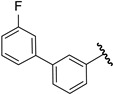	6.32	6.54	0.22	6.51	0.19
**26**	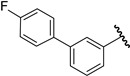	7.03	6.86	−0.17	6.81	−0.22
**34**	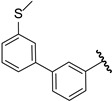	6.82	6.81	−0.01	7.15	0.32
**35**	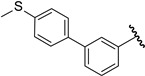	6.85	7.40	0.55	6.82	−0.03

**Table 2:** Internal and external validation results of CoMFA and CoMSIA models.

Statistical Parameters	CoMFA	CoMSIA
q^2^ *^a^*	0.626	0.605
N *^b^*	8	6
r^2^ *^c^*	0.991	0.964
SEE *^d^*	0.090	0.173
F *^e^*	327.630	116.389
r^2^_pred_ *^f^*	0.941	0.961
r^2^_m_ *^g^*	0.796	0.919
SDEP_ext_ *^h^*	0.260	0.308
Fraction of field contributions		
S *^i^*	0.729	0.163
E *^j^*	0.271	0.228
D *^k^*	-	0.222
A *^l^*	-	0.387

*^a^* Cross-validation correlation coefficient (q^2^). *^b^* The optimal number of components (N). *^c^* Coefficient of determination (r^2^). *^d^* Standard error of estimate (SEE). *^e^* F-test value (F). *^f^* External validation determination coefficient (r^2^_pred_). *^g^* Modified r^2^ term (r^2^_m_). *^h^* External standard deviation error of prediction (SDEP_ext_). *^i^* The steric field (S). *^j^* The electrostatic field (E). *^k^* The hydrogen bond donor field (D). *^l^* The hydrogen bond acceptor field (A).

**Table 3:** Newly designed anti-SKOV3 compound **42**, its corresponding pred. and exp. pIC_50_ activity values.

Comp.	Structure	Pred. (CoMFA)	Pred. (CoMSIA)	Exp. (pIC_50_)
**42**	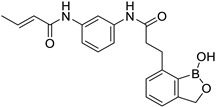	7.19	7.34	7.40

## Data Availability

The original contributions presented in this study are included in the article/Supplementary Materials. Further inquiries can be directed to the corresponding authors.

## Supplementary Materials

The following supporting information can be downloaded at https://www.mdpi.com/article/10.3390/ijms27010472/s1.
