# Supplementary material for: Discovery of New 7-Propanamide Benzoxaborole as Potent Anti-SKOV3 Agent via 3D-QSAR Models
Source: Int J Mol Sci. 2026 Jan 2;27(1):472. doi: 10.3390/ijms27010472 (PMC12786075; doi:10.3390/ijms27010472)
Supplement: Supplementary file 1 [file ijms-27-00472-s001.zip › ijms-4027348-supplementary.pdf]

Discovery of New 7-Propanamide Benzoxaborole as Potent Anti-SKOV3 Agent via 3D-QSAR Models

Liyang Ji [1,2], Jiong Zhang [3], Huchen Zhou [2,*] and Yaxue Zhao [1,*]

[1] School of Pharmaceutical Sciences, Shanghai Jiao Tong University, Shanghai 200240, China; lyji2020@sjtu.edu.cn
[2] Key Laboratory of Tropical Biological Resources of Ministry of Education and Hainan Engineering Research Center for Drug Screening and Evaluation, School of Pharmaceutical Sciences, Hainan University, Haikou 570228, China
[3] Inflammation and Immune Mediated Diseases Laboratory of Anhui Province, School of Pharmacy, Anhui Medical University, Hefei 230032, China; zhangjiong@ahmu.edu.cn
* Correspondence: hczhou@hainanu.edu.cn (H.Z.); yaxuezhao@sjtu.edu.cn (Y.Z.)

Supporting information

Table of Contents

I. NMR spectra of amine intermediates 45 and 46

II. NMR spectra of 7-propanamide benzoxaborole 42

III. HPLC spectrum of 7-propanamide benzoxaborole 42

IV. Mass spectra of 7-propanamide benzoxaborole 42

V. Cell level data

VI. Statistical parameters of QASR modeling using alternative conformers (Tables S1 and S2)

I. NMR spectra of amine intermediates 45 and 46

Compound **45**

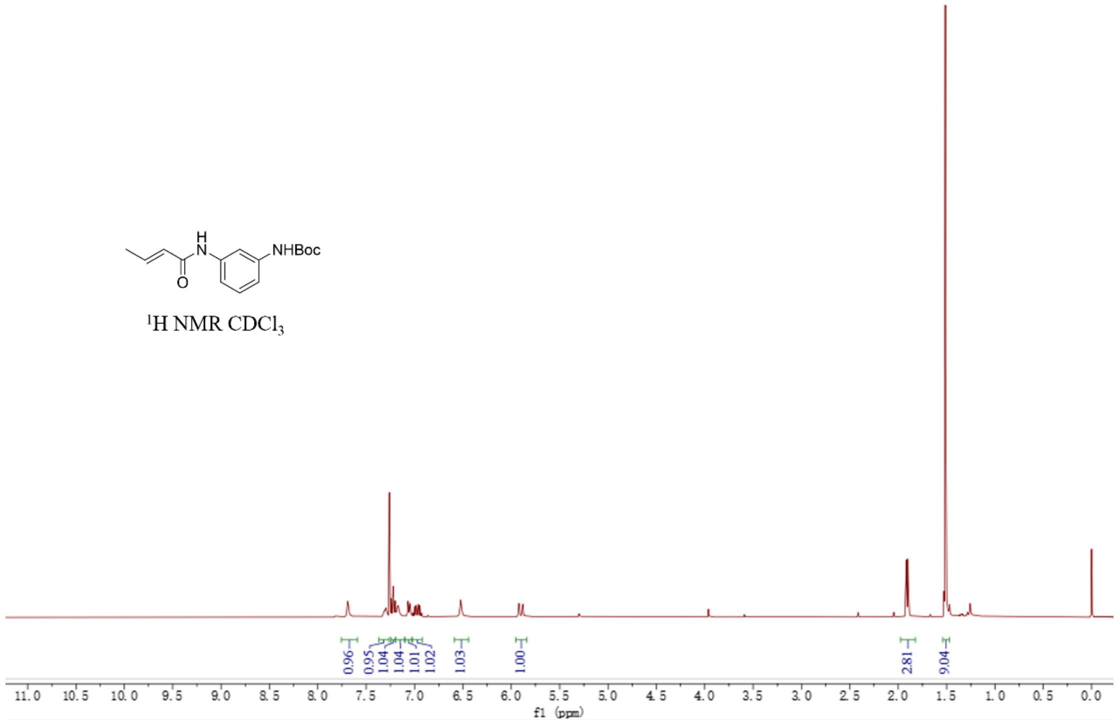

Compound **46**

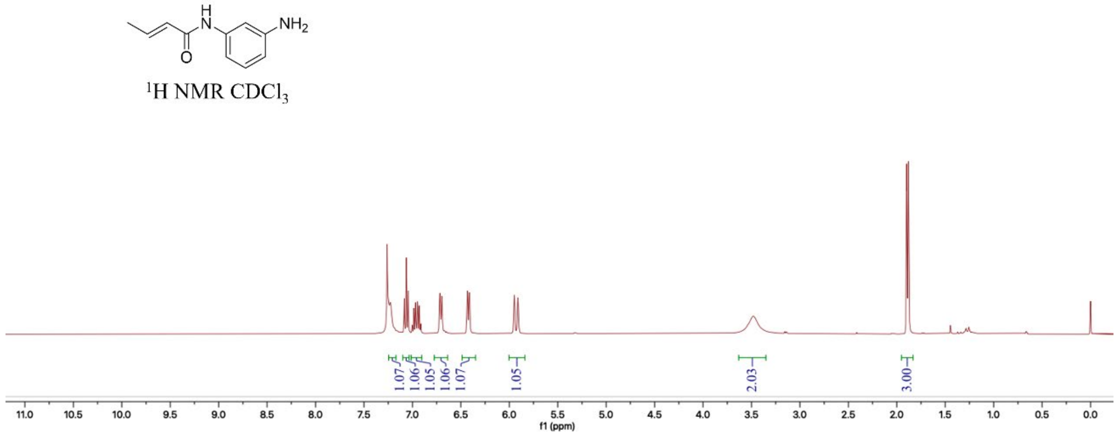

II. NMR spectra of 7-propanamide benzoxaborole 42

Compound **42**

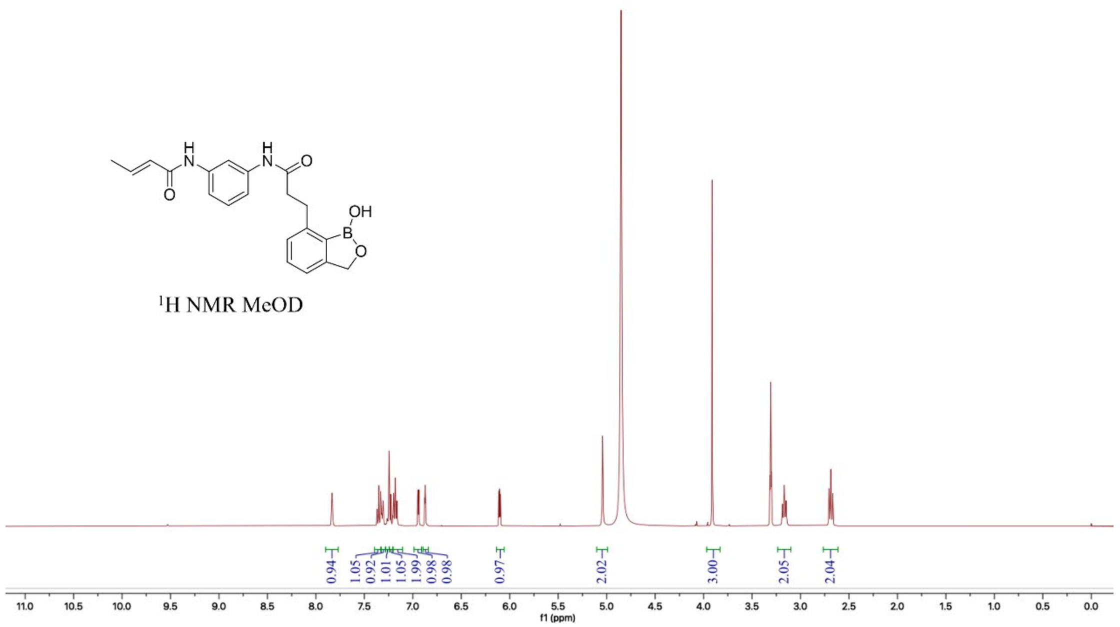

Compound **42**

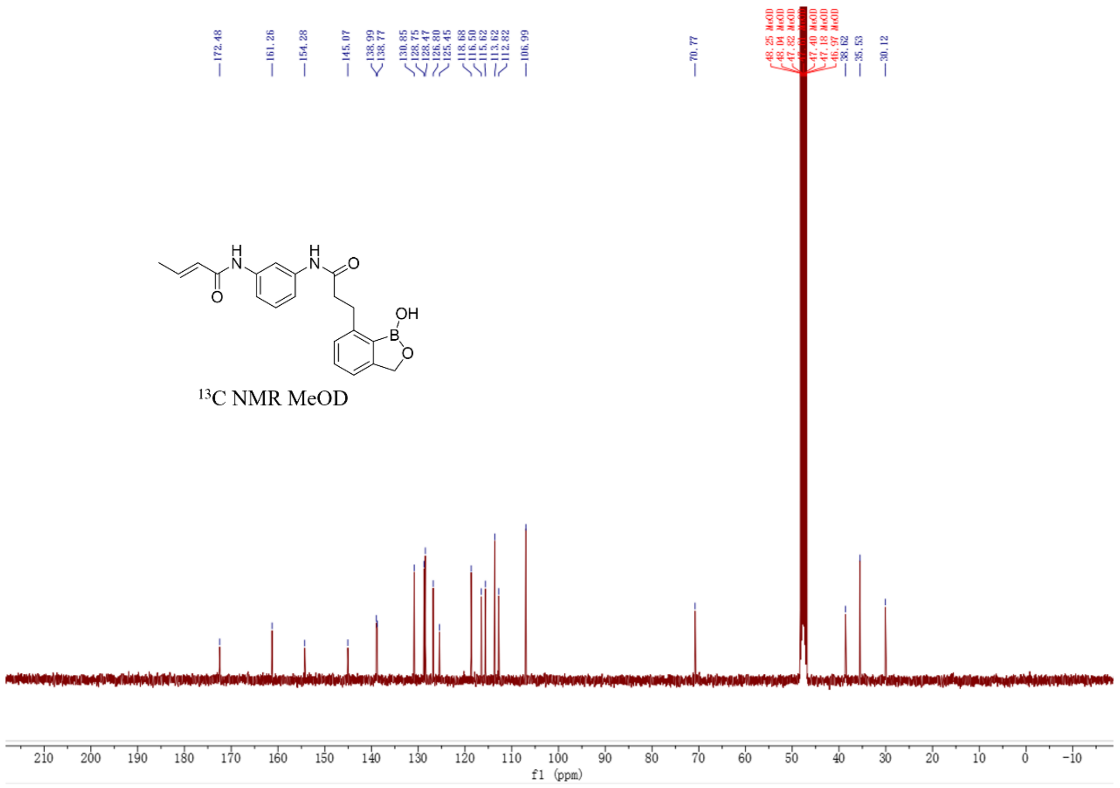

III. HPLC spectrum of 7-propanamide benzoxaborole 42

Compound **42**

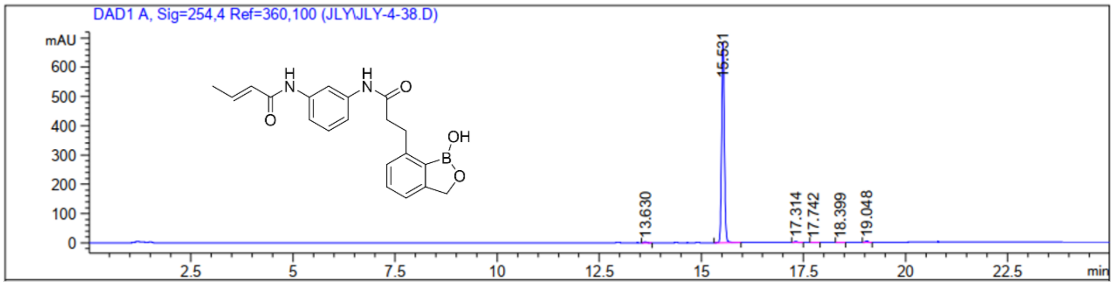

信号 1: DAD1 A, Sig=254,4 Ref=360,100

峰 #	保留时间 [min]	类型	峰宽 [min]	峰面积 [mAU*s]	峰高 [mAU]	峰面积 %
1	13.630	BB	0.0713	14.71789	3.10251	0.4536
2	15.531	BB	0.0721	3184.66992	686.61432	98.1582
3	17.314	VB	0.0733	17.58937	3.71155	0.5421
4	17.742	BB	0.0717	4.07186	8.85174e-1	0.1255
5	18.399	BB	0.1039	3.78934	5.67856e-1	0.1168
6	19.048	BB	0.0650	19.58684	4.66592	0.6037
总量 :				3244.42523	699.54733	

IV. Mass spectra of 7-propanamide benzoxaborole 42

Compound **42**

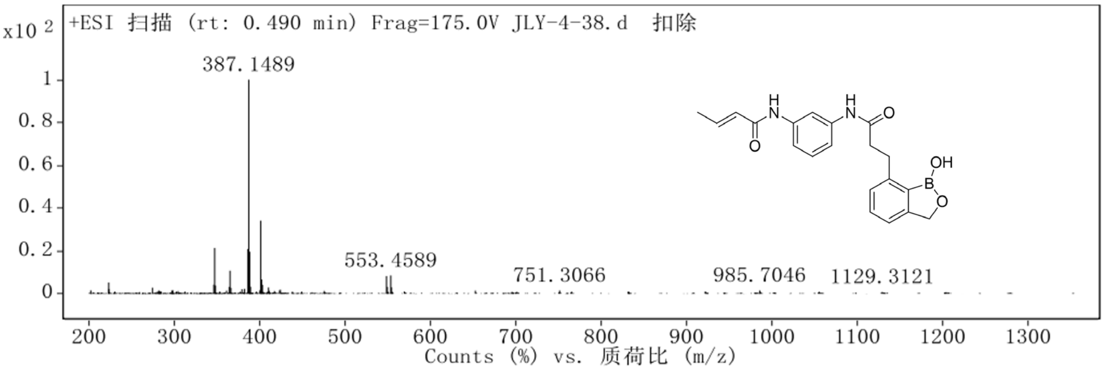

V. Cell level data

Compound **42**

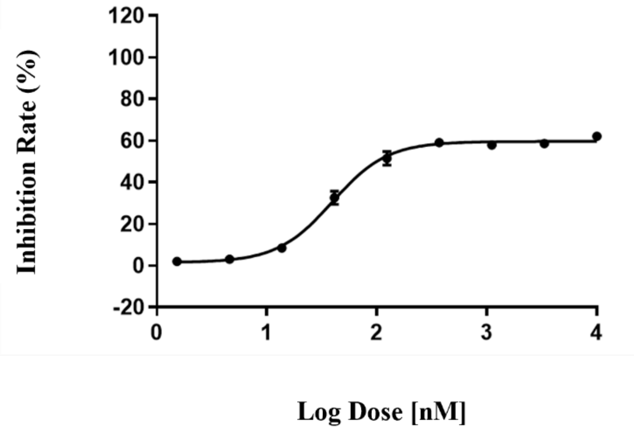

SKOV3 IC_{50} = 39.4 nM

VI. Statistical parameters of QASR modeling using alternative conformers (Tables S1 and S2)

Table S1. Internal and external validation results of CoMFA and CoMSIA models by conformational ensemble 2.

Statistical parameters	CoMFA	CoMSIA
q^{2a}	0.324	0.346
N^{b}	4	3
r^{2c}	0.943	0.846
SEE^{d}	0.211	0.339
F^{e}	114.987	52.971
$r^2_{pred}{}^{f}$	/	/
$r^2_{m}{}^{g}$	/	/
$SDEP_{ext}{}^{h}$	/	/
**Fraction of field contributions**		
Steric	0.742	0.127
Electrostatic	0.258	0.131
Hydrogen bond donor	/	0.369
Hydrogen bond acceptor	/	0.373

^{a}Cross-validation correlation coefficient (q^2). ^{b}The optimal number of components (N). ^{c}Coefficient of determination (r^2). ^{d}Standard error of estimate (SEE). ^{e}F-test value (F). ^{f}External validation determination coefficient (r^2_{pred}). ^{g}Modified r^2 term (r^2_{m}). ^{h}External standard deviation error of prediction ($SDEP_{ext}$).

Table S2. Internal and external validation results of CoMFA and CoMSIA models by conformational ensemble 3.

Statistical parameters	CoMFA	CoMSIA
q^{2a}	0.076	0.186
N^{b}	1	5
r^{2c}	0.435	0.962
SEE^{d}	0.628	0.174
F^{e}	23.865	137.306
$r^2_{pred}{}^{f}$	/	/
$r^2_{m}{}^{g}$	/	/
$SDEP_{ext}{}^{h}$	/	/
**Fraction of field contributions**		
Steric	0.716	0.135
Electrostatic	0.284	0.229
Hydrogen bond donor	/	0.261
Hydrogen bond acceptor	/	0.375

[a]Cross-validation correlation coefficient (q^2). [b]The optimal number of components (N). [c]Coefficient of determination (r^2). [d]Standard error of estimate (SEE). [e]F-test value (F). [f]External validation determination coefficient (r^2_{pred}). [g]Modified r^2 term (r^2_m). [h]External standard deviation error of prediction ($SDEP_{ext}$).
